# Implementation of a Biopsychosocial History and Physical Exam Template in the Electronic Health Record: Mixed Methods Study

**DOI:** 10.2196/42364

**Published:** 2023-02-21

**Authors:** Erin Y Rieger, Irsk J Anderson, Valerie G Press, Michael X Cui, Vineet M Arora, Brent C Williams, Joyce W Tang

**Affiliations:** 1 Department of Internal Medicine Columbia University Medical Center New York, NY United States; 2 Department of Medicine University of Chicago Chicago, IL United States; 3 Department of Internal Medicine Rush University Chicago, IL United States; 4 Department of Internal Medicine University of Michigan Ann Arbor, MI United States

**Keywords:** medical education, electronic health record, hospital medicine, psychosocial factors, chronic condition, chronic, disease, management, prevention, clinical, engagement

## Abstract

**Background:**

Patients’ perspectives and social contexts are critical for prevention of hospital readmissions; however, neither is routinely assessed using the traditional history and physical (H&P) examination nor commonly documented in the electronic health record (EHR). The H&P 360 is a revised H&P template that integrates routine assessment of patient perspectives and goals, mental health, and an expanded social history (behavioral health, social support, living environment and resources, function). Although the H&P 360 has shown promise in increasing psychosocial documentation in focused teaching contexts, its uptake and impact in routine clinical settings are unknown.

**Objective:**

The aim of this study was to assess the feasibility, acceptability, and impact on care planning of implementing an inpatient H&P 360 template in the EHR for use by fourth-year medical students.

**Methods:**

A mixed methods study design was used. Fourth-year medical students on internal medicine subinternship (subI) services were given a brief training on the H&P 360 and access to EHR-based H&P 360 templates. Students not working in the intensive care unit (ICU) were asked to use the templates at least once per call cycle, whereas use by ICU students was elective. An EHR query was used to identify all H&P 360 and traditional H&P admission notes authored by non-ICU students at University of Chicago (UC) Medicine. Of these notes, all H&P 360 notes and a sample of traditional H&P notes were reviewed by two researchers for the presence of H&P 360 domains and impact on patient care. A postcourse survey was administered to query all students for their perspectives on the H&P 360.

**Results:**

Of the 13 non-ICU subIs at UC Medicine, 6 (46%) used the H&P 360 templates at least once, which accounted for 14%-92% of their authored admission notes (median 56%). Content analysis was performed with 45 H&P 360 notes and 54 traditional H&P notes. Psychosocial documentation across all H&P 360 domains (patient perspectives and goals, mental health, expanded social history elements) was more common in H&P 360 compared with traditional notes. Related to impact on patient care, H&P 360 notes more commonly identified needs (20% H&P 360; 9% H&P) and described interdisciplinary coordination (78% H&P 360; 41% H&P). Of the 11 subIs completing surveys, the vast majority (n=10, 91%) felt the H&P 360 helped them understand patient goals and improved the patient-provider relationship. Most students (n=8, 73%) felt the H&P 360 took an appropriate amount of time.

**Conclusions:**

Students who applied the H&P 360 using templated notes in the EHR found it feasible and helpful. These students wrote notes reflecting enhanced assessment of goals and perspectives for patient-engaged care and contextual factors important to preventing rehospitalization. Reasons some students did not use the templated H&P 360 should be examined in future studies. Uptake may be enhanced through earlier and repeated exposure and greater engagement by residents and attendings. Larger-scale implementation studies can help further elucidate the complexities of implementing nonbiomedical information within EHRs.

## Introduction

The posthospitalization period is a particularly vulnerable time for patients as they may need to adjust to new or evolving diagnoses, modify medication regimens, navigate new mobility limitations, and implement new lifestyle changes. To facilitate care transitions, reinforce chronic disease management, and prevent rehospitalization, it is essential to understand patient perspectives as well as patients’ unique social context. The presence of unmet social needs has been strongly linked with inferior health outcomes [1] as well as a higher risk of rehospitalization [2-4]. Despite this connection, patient perspectives and social context are not systematically assessed with the traditional history and physical (H&P) examination. While the World Health Organization supports inclusion of social, economic, and political aspects of health in the training of medical students globally [5], and the Institute of Medicine has recommended collection of social determinants of health in the electronic health record (EHR) [6], psychosocial documentation remains limited. The fundamental history-taking framework through which physicians approach diagnosis and management has changed very little over the past 50 years. Physician documentation still focuses more on biomedical domains rather than on the psychosocial context [7,8]. Consequently, graduating medical students and residents are not prepared to ask critical questions related to the psychosocial context [9].

The H&P 360 is a revised template for conducting an H&P that applies a 7-domain biopsychosocial framework, integrating assessment of patient perspectives and goals, mental health, and an expanded social history (behavioral health, social support, living environment and resources, and functional status) with collection of biomedical information (see Multimedia Appendix 1) [10]. This template has shown promise in increasing psychosocial documentation in standardized evaluation settings, including a one-time-point use in an inpatient subinternship (subI) [10] and an Objective Structured Clinical Examination exercise during which third- and fourth-year medical students were randomized to use of the H&P 360 or the standard H&P [11]. However, potential uptake and impact with more routine use of the H&P 360 in usual clinical teaching settings remain unknown.

Given the ubiquity and ease of developing templated notes to facilitate documentation within the EHR among practicing clinicians, residents, and students in many countries, creation of a templated note guiding students through the H&P 360 domains could be one approach to promote uptake. EHR-based template studies to date have focused on inpatient resident and faculty subjects with primary endpoints including note quality, quality of care, and time to note completion [12-14]. However, few studies have evaluated the use of templates to intentionally improve documentation related to patient perspectives and social and behavioral determinants of health [15,16]; most such initiatives have centered on interprofessional team members rather than on promoting psychosocial documentation within a physician’s scope of work [17,18].

The objective of this study was to assess the feasibility, acceptability, and impact on care planning of implementing an inpatient H&P 360 template in the EHR for use by fourth-year medical students during their internal medicine subI.

## Methods

### Study Design

This implementation study included fourth-year medical students (MS4) completing their internal medicine inpatient subI at the University of Chicago (UC) during the 2020-2021 academic year. The evaluation plan was based on the Kirkpatrick model (Reaction, Learning, Behavior, Organizational Performance), building on prior work with standardized patients (“can do”) to use in usual clinical settings (“does”) [19]. Student reaction and learning were assessed through a postintervention survey. Behavioral change and organizational performance were assessed by measuring utilization of the EHR template and through content analysis of student notes.

### Ethics Approval

The UC Institutional Review Board granted the student survey an exemption under quality improvement status. The UC Institutional Review Board (IRB19-1800; IRB21-0571) granted exemption approval for the review and qualitative analysis of student clinical notes. A waiver of informed consent was granted due to the retrospective design and patients and students being unavailable for consent.

### EHR Template Development

A team composed of 2 general internists (one of whom was the course director for the internal medicine subI) and 2 hospital medicine physicians (one of whom was also a bioinformatics fellow) adapted the H&P 360 for use with inpatients and created the EHR templates [11]. The full H&P 360 template included components of a traditional H&P with expanded sections specific to the H&P 360 domains (see Multimedia Appendix 2). Under history of present illness, the template included prompts for: (1) patient understanding of health, (2) self-assessed control, (3) patient-identified strengths, (4) patient-identified barriers, (5) patient priorities and goals, and (6) psychosocial problems and concerns. Under social history, the template included prompts for documentation under the following domains: (1) behavioral health, (2) social support, (3) living environment and resources, and (4) function. Finally, under the assessment and plan, in addition to the typical headings prompting documentation of evaluation and management of acute and chronic biomedical problems, there was an added heading for interdisciplinary resource needs.

The team engaged a group of 4 fourth-year medical students participating in internal medicine subIs to pilot various iterations of the template to improve usability. Based on feedback from the students, who desired maximum flexibility with documentation, the decision was made to allow free-text responses under each domain rather than using drop-down response options. In addition, while some students preferred to use a full de novo H&P 360 template, others preferred to insert unique H&P 360 elements into other existing templates. As a result, two templates were created to accommodate flexibility in documentation: one that could be used as a complete H&P and another that allowed integration of only the unique H&P 360 domains into any H&P template or progress note. Students also suggested that we create a visual reminder for the H&P 360 domains that could be referenced during history-taking; based on this feedback, we created and offered cards for student ID badges listing the H&P 360 domains and relevant content areas.

### Participants

UC Pritzker School of Medicine fourth-year medical students participate in a 4-week inpatient subI of their choosing. SubIs in internal medicine choose to rotate in general internal medicine, clinical cardiology, or the medical intensive care unit (ICU) (all at UC) or at an offsite community hospital teaching affiliate. SubIs are on call every 3-4 days and may admit up to three patients per call day.

Between August 2020 and April 2021, 24 internal medicine subIs were enrolled in the H&P 360 educational program. Prior to their subI month, students received an orientation email from the course director (author IJA) describing the H&P 360 model and providing the note templates, smart phrases for pulling up the templates, and use expectations. The two H&P 360 templates were shared with the students via the EHR. One could be used as a full H&P note template (Multimedia Appendix 2). The second template contained only the elements unique to the H&P 360 and excerpts could be merged into any traditional H&P template (Multimedia Appendix 3). SubIs were asked to use one of these H&P 360 templates in at least one admitting note per call cycle. Students also received cards for their ID badge listing the H&P 360 domains to reference during the patient encounter. The on-service attending physicians were informed of the expectations via email and provided with informational materials about the H&P 360 and rationale for use. During a monthly subI noon conference with author IJA, students were invited to informally discuss their experience using the H&P 360 template.

### Utilization of the H&P 360 Template

H&P 360 template usage was measured to understand its feasibility and acceptability. Research coordinators conducted an EHR query to retrospectively identify all admission notes written by students during their subI in general internal medicine or cardiology at UC during the 2020-2021 academic year (n=13 students). Notes written by subIs in the ICU were excluded because of expected admission note differences in this setting and competing priorities for ICU patients at admission. SubIs at the affiliate health care system conducted documentation in a separate, inaccessible EHR, thereby precluding collection of their notes. The research coordinators identified all subI admission notes utilizing an H&P 360 template; all other admission notes were labeled as utilizing traditional (ie, any non-H&P 360) templates. The proportion of all notes written using the H&P 360 template was calculated per student and in total.

### Content Analysis

Content analysis was performed to assess the impact of the H&P 360 template. For purposes of qualitative comparison of note content, research coordinators collected and deidentified all of the H&P 360 notes and a sample of the traditional notes. The sample of traditional notes was drawn by attempting relatively balanced representation across students. Specifically, each student could contribute no more than 5 traditional notes to the total sample; for those with more than 5 traditional notes, a random subsample was selected for inclusion.

The content analysis team was composed of three internists involved in medical education (JWT, VGP, IJA) and one medical student (EYR). Throughout the process of analysis, team members discussed their preconceived notions and biases from their roles in education and patient care. The team began with a set of a priori content domains based on the H&P 360 template (eg, mental health, behavioral health, social support). The team members independently reviewed a set of notes—four from the H&P 360 group and four from the traditional group—to clarify the definition of the content domains, add additional de novo content domains as needed, and improve consistency between coders. Subsequently, for each of the notes, two team members extracted relevant text and entered it into a Research Electronic Data Capture (REDCap) template under the appropriate content domain. Discrepancies in coding were reviewed and resolved through email correspondence. The text from each content domain was then aggregated into a document and reviewed by two members of the team to identify themes within each content domain and to assess whether there were qualitative differences in the content between the H&P 360 and traditional templated notes. Each content domain was discussed at the weekly group video meeting. The number of notes categorized under each content domain was counted for the H&P 360 and traditional templated groups.

### Student Survey

A student survey was used to assess student perceptions of feasibility, acceptability, and impact of the H&P 360. Survey items assessing student perception of the H&P 360 were developed in collaboration with the American Medical Association H&P 360 Implementation Grantee team. The survey consisted of 14 5-point Likert-scale questions assessing feasibility, perceived impact on patient care, and perceived impact on educational experience. Short-response items elicited useful and challenging aspects of the H&P 360 and student recommendations (Multimedia Appendix 4).

At the conclusion of the educational program, all subIs (n=24 students) were asked to complete the survey anonymously. Percentages of students who selected 5 (strongly agree) or 4 (somewhat agree) on the Likert scale were tabulated. Open-ended responses were read by two members of the research team and common statements (defined as reported by three or more students) were identified and summarized.

## Results

### Utilization of the H&P 360 Template

Utilization of the H&P 360 could be directly measured among the 13 subIs rotating on the UC general medicine or cardiology services during the 2020-2021 academic year. This group authored a total of 164 admission notes in the EHR. Of all admission notes, 45 (27.4%) were written with an H&P 360 template (Multimedia Appendix 5). As mentioned above, subIs rotating in the ICU or at the community hospital teaching affiliate were excluded from this analysis.

Of the 13 subIs, 6 (46%) students authored at least one admission note using an H&P 360 template. These H&P 360 templated notes accounted for 14%-92% of their authored admission notes (median 56%). Seven students (54%) never authored a note using the H&P 360 templates.

### Content Analysis

Content analysis was performed with 45 H&P 360 notes and 54 traditional H&P notes (Table 1).

**Table 1:** Documentation of content domains across the history and physical (H&P) 360 and traditional H&P notes.

Content domain		H&P 360 notes (n=45), n (%)	Traditional H&P notes (n=54), n (%)
**Patient perspectives and mental health**			
	Patient understanding of health	23 (51)	16 (30)
	Patient priorities and goals	18 (40)	4 (7)
	Mental health	15 (33)	8 (15)
**Expanded social history**			
	Behavioral health (nonsubstance use)	32 (71)	23 (43)
	Social support	44 (98)	28 (52)
	Living environment and resources	16 (36)	10 (19)
	Function	42 (93)	31 (57)
**Impact on patient care**			
	Needs identified	9 (20)	5 (9)
	Education and counseling	12 (27)	13 (24)
	Interdisciplinary resource coordination	35 (78)	22 (41)

### Patient Perspectives and Mental Health

#### Patient Perspectives

Text was coded for patient understanding of health and patient priorities or goals. Some H&P 360 notes retained and responded directly to the EHR template prompts for these elements within the patient subjective history, while others spontaneously integrated this content into other areas of the note.

While 7% (4/54) of traditional notes documented patient priorities or goals, this was documented in 40% (18/45) of H&P 360 templated notes. Qualitative differences between groups were also identified in the content coded for this domain. H&P 360 notes discussed priorities related to decreasing pain, increasing function, determining the cause of one’s symptoms, wanting to improve chronic disease management, and wanting to go home.

Patient priorities and goals: Would like to make sure no underlying etiology of current a-fib [atrial fibrillation] episode. Pt [patient] reports h/o [history of] diagnosis of T2DM [type 2 diabetes mellitus] and is trying to improve w/ lifestyle modification and does not like to take medications.

H&P 360 note, Student F

In contrast, for traditional notes, the only documented priorities or goals related to the patient wanting to leave the hospital: “She wants to go home.” [Traditional note, Student N]

Patient understanding of health was documented in 51% (23/45) of H&P 360 notes and in 30% (16/54) of traditional notes. Documentation across both groups related to patient perceptions as to the cause of their symptoms or patient familiarity with their medications: “He states he has recurrent episodes of Afib [atrial fibrillation] since 2013 w/ similar symptoms (he has a watch that alerts him).” [Traditional note, Student K]

Among H&P 360 notes, some also included information from the perspective of the patient or clinician of the patient’s level of understanding of their diagnoses, medications, or disease etiology.

Patient understanding of health: Pt [patient] understands the reason that she required her extensive surgery, and she has a clear understanding of the reasons for her various medications. Patient self-assessed control: Pt reports feeling like her health status is currently “out of [her] control.” She states that her health is “in the lord’s hands.”

H&P 360 note, Student C

#### Mental Health

Overall, 33% (15/45) of H&P 360 notes and 15% (8/54) of traditional notes included the mental health domain. Across both groups, there was documentation regarding psychiatric diagnoses and related treatment, anxiety, stress, substance use, or documentation that there were no relevant concerns in this domain. Qualitative differences between groups were not identified. One such example was: “...increased stress related to her brother’s condition and the need to pay for his medical expenses.” [Traditional note, Student G]

### Expanded Social History

#### Behavioral Health (Nonsubstance Use)

A majority of notes in both groups contained autopopulated text related to tobacco, alcohol, and drug use. Since it was unclear whether this information was input by the author of the note or had been documented in the EHR from a prior encounter, this information was not included for the purposes of this analysis. The behavioral health domain (excluding information about tobacco, alcohol, and drug use) was present in 71% (32/45) of H&P 360 notes versus 43% (n=23/54) of traditional notes. Across both groups, text coded for behavioral health frequently documented patient adherence to medications. Physical activity and nutrition behaviors were also described across both groups. Qualitative differences in the coded text were not identified: “States takes meds regularly and doesn’t miss... States his wife cooks-does not use salt. Does little physical activity like stairs.” [H&P 360 note, Student D]

#### Social Support

Information about the patient’s social network was documented in 98% (44/45) of H&P 360 notes and in 52% (28/54) of traditional notes. The social support domain included information about the patient’s cohabitants, other important relationships, and presence of home health workers. Across both groups, there was also information about how the patient’s social network assisted in their care. No qualitative differences were observed in the coded text: “The patient currently lives with her daughter. her medications are managed at home by her son, who is a nurse.” [Traditional note, Student A]

#### Living Environment and Resources

Overall, 36% (16/45) of H&P 360 notes and 19% (10/54) of traditional notes documented information about patient’s access to housing, transportation, food, insurance, or financial resources. The coded content was qualitatively similar across both groups.

Previously living with friend, but patient denies living with anyone currently. Does not offer further details of living arrangements. Patient seems to be living independently, but is not clear as to whether she is living with others or receives help.

H&P 360 note, Student G

#### Function

Patient functioning prior to hospitalization was documented in 93% (42/45) of H&P 360 templated notes and in 57% (31/54) of traditional notes. Across both groups, this domain was qualitatively similar. Both groups documented activities of daily living, instrumental activities of daily living, mobility, assistive devices, cognitive functioning, and occupation.

At baseline, patient uses powerchair for mobility since 2010. She is able to eat and use the bathroom on her own but requires assistance to cook, clean, shower, and do her home leg therapy.

H&P 360 note, Student H

### Impact on Patient Care

#### Needs Identified

Resource needs were identified in 20% (9/45) of H&P 360 templated notes and in 9% (5/54) of traditional notes. Needs were commonly related to placement due to concerns about safety, insufficient caregiving in the home setting, or housing instability. Identified needs also commonly included insurance issues, medication refills, or outpatient follow up. In qualitative comparison, text from the H&P 360 notes contained more detail about resource needs. Plans for addressing needs were usually but not always explicitly described. The plans often involved acquiring equipment or involving social work. In situations where a plan was not stated, it was unclear if it was assumed that it would be addressed or if it ultimately was not addressed.

Per pt’s [patient’s] niece, concern for abuse and neglect at pt’s home. Pt endorses verbal abuse/threats from daughter, denies any physical harm. - SW [social work] following, contacted elder abuse hotline, case assigned to Center for New Horizons who will f/u [follow up] with pt and family members.

H&P 360 note, Student C

Needs PCP [primary care provider]- no insurance, SW [social work] consult to help establish with Medicaid.

Traditional note, Student L

#### Education and Counseling

Patient education or counseling was described in 27% (12/45) of H&P 360 notes and in 24% (13/54) of traditional notes. Across both groups, documented counseling most often involved nutrition, physical activity, and substance use, while some notes documented patient education regarding management options. There was little detail in excerpts from either group. No qualitative differences were identified: “Encourage elevation of legs during sitting and during bedtime. Compression stockings as outpatient.” [Traditional note, Student O]

#### Interdisciplinary Resource Coordination

Interdisciplinary resource coordination was documented in 78% (35/45) of H&P 360 notes and in 41% (22/54) of traditional notes. This code included inpatient and outpatient referrals to social work, physical or occupational therapy, nutrition, podiatry, and medical specialties. Across both groups, a majority of the documentation was simply noting that physical or occupational therapy services were ordered for the patient. There was not much detail in either group. Qualitative differences were not identified: Social work consulted on prior admission. Consider referral for inpatient vs outpatient rehab services. [Traditional note, Student C].

### Student Survey

Of all subIs in internal medicine (N=24), 11 (45%) completed the survey regarding their experience with the H&P 360 (Table 2). Regarding feasibility of the H&P 360, the majority of respondents strongly or somewhat agreed that the H&P 360 took an appropriate amount of time to complete and strongly or somewhat agreed that it was easy to use. However, few respondents strongly or somewhat agreed that presentations using the H&P 360 were well-received by the clinical team. Regarding perceived impact on patient care, respondents strongly or somewhat agreed that the H&P 360 helped them better understand patient goals, facilitated a stronger provider-patient relationship, changed some of the questions they asked during the encounter, and added valuable information that they would not have known about the patient. Few students strongly or somewhat agreed that the H&P 360 helped them to create a more comprehensive problem list (Table 2).

**Table 2:** Survey respondents somewhat agreeing or strongly agreeing with statement (N=11).

Statement regarding H&P^a^ 360		Agree with statement^b^, n (%)
**Feasibility**		
	Took an appropriate amount of time to complete	8 (73)
	Was easy to use	7 (64)
	Could be incorporated into every patient interaction	6 (55)
	Presentations were well-received by my clinical team	3 (27)
**Perceived impact on patient care**		
	Helped me better understand patients’ goals	10 (91)
	Facilitated a stronger provider-patient relationship	9 (82)
	Changed some of the questions I ask patients during the encounter	10 (91)
	Added valuable information that I would not otherwise know about the patient	9 (82)
	Facilitated care planning that included other health professionals	7 (64)
	Helped improve the care I provided to my patients	6 (55)
	Was able to incorporate information into management plans	5 (45)
	Helped create a more comprehensive problem list	4 (36)
**Perceived impact on education**		
	Helped me learn to be a better clinician	7 (64)
	Plan to use elements during other rotations	7 (64)

^a^H&P: health and physical.

^b^Survey prompts were answered on a 5-point Likert scale. Responses were dichotomized as agreeing with statement if 5 (strongly agree) or 4 (somewhat agree).

In open-ended prompts on the survey, five students shared that the H&P 360 served as a prompt to further explore or document social history. One student wrote:

It provided examples for what to ask in order to learn more information about patient’s social and home life...It alerted me to important things that we often don’t ask or miss when taking care of inpatients.

Three students stated that the template helped them clarify patient goals.

Regarding areas for improvement, four students noted the time that it took to complete the H&P 360. One of these students recommended having the option for shorter, drop-down answers available in the template.

Three students shared that they thought patients were surprised to be asked about some of the topics covered in the H&P 360. One student wrote:

I think the biggest challenge is that patients aren’t used to being asked some of these questions (their goals, their self-perceptions of their health) during these admissions. It can be a delicate subject.

Finally, three students reported concerns about deviating from the note template typically used on their clinical service. Two students specifically reported receiving negative feedback from their clinical team. One wrote:

...at times I would get feedback from my residents that they wished the information was incorporated elsewhere. It was also cumbersome to be expected to document so much info that oftentimes is nice and useful to know, but that my team did not necessarily want to hear about.

## Discussion

### Principal Findings

In this inpatient implementation study of the H&P 360 EHR template, psychosocial documentation was more common across virtually all H&P 360 content domains among admission notes using the H&P 360 template compared to the traditional H&P note template. Importantly, documentation was also more common with respect to social needs identification and interdisciplinary collaboration. However, the overall impact of the tool was diminished by limited and variable uptake of the H&P 360 note template by the subI students.

While students generally provided positive feedback about the potential of the H&P 360 to improve understanding of patient goals and to enhance the patient-provider relationship, students less often reported that this added information changed treatment plans or improved care. There are several potential reasons for this apparent paradox. First, students are already including health-related social needs in care planning closer to the time of discharge (not captured in admission notes). Alternatively, they gather information but do not apply it (potentially due to barriers related to time, resources, or interdisciplinary support).

Many students did not use the H&P 360 template. Open-ended survey feedback suggested that a barrier to use may be the time required to complete the expanded H&P. Drop-down menu responses could increase ease of template use; however, these may also limit detailed communication of the patient’s context or preferences. Pacing collection of psychosocial information throughout the hospital stay beyond the admission day, perhaps through triggered alerts or reminders, could decrease and spread out the time required; this pacing may in some cases improve perceived relevance and acceptability to students and patients as acute biomedical issues have abated.

In addition to time constraints, several students also noted negative feedback from some team members who felt that the psychosocial information presented within the context of the H&P 360 appeared to deviate from expected convention. Students have strong incentives to assimilate with their team and thus likely felt pressure not to use the H&P 360 template even if they found it useful. The lack of interest among other team members in patients’ contextual information likely relates in part to the historical focus physicians have had on biomedical information. Further, the timing of presenting this information may have been a factor as students’ perceptions of the relative value of this contextual information may be lower in informing initial treatment and stabilization plans at admission as compared with the longer-term planning that occurs nearer to discharge.

This pragmatic implementation provided only a low-intensity orientation to the H&P 360 for faculty in the form of emailed materials. Future efforts will need to increase and improve orientation of faculty to the H&P 360 as well as include training for resident physicians. Student uptake of the H&P 360 EHR template may be further enhanced through exposure in the preclinical years in settings such as free clinics and clinical preceptor groups.

### Comparison With Prior Work

To date, EHR tools and templates have predominantly been leveraged to enhance biomedical documentation, targeting quality metrics, and optimizing reimbursement [13,20-22]. Our study represents an important contribution to this literature as there is limited research on use of EHR templates to improve psychosocial documentation or to intentionally elicit patients’ perspectives and goals. Several initiatives in the United States call for improved integration of screening of social determinants of health into health care delivery and the need for standardized methods for capturing this information in EHRs [6,23,24]. Systematic documentation of patients’ needs and goals during hospitalization has the potential to not only improve the care of individual patients (personalizing care, supporting shared decision-making, aiding discharge planning), but can provide critical context for health systems in designing programs and determining staffing needs to meet the needs of the patient population they serve [23,25].

While most interventions to promote psychosocial documentation in the EHR have focused on the completion of expanded checklists and screening tools primarily by nonphysician team members, we intentionally chose to include psychosocial documentation within the physician note template [17,18,26]. This choice was made to match the usual workflow for students and residents at our institution and to promote this documentation as a part of the physician’s sphere of work (rather than an area delegated to social workers, nurses, or others).

While EHR templates have been found to improve documentation of key measures, some studies suggest that this may occur at the expense of patient-centered care, prioritizing the clinician’s agenda above that of the patient [27]. However, in contrast to many EHR templates, the H&P 360 promotes a domain-based approach to discussing psychosocial concerns with patients (rather than a checklist-based approach) and further intentionally solicits patients’ goals and perspectives. Integration of patient-centered questions within templates used by general practitioner practices in England was actually found to increase the perception of patient-centeredness [16].

### Limitations

There are several important limitations to note. First, while we found that psychosocial documentation was more common in the H&P 360 notes as compared with traditional notes, our study design did not allow for rigorous statistical testing. Second, the low and variable uptake of the EHR template meant that our sample of representative H&P 360 notes was drawn from a small number of students, thus limiting the generalizability of our findings. Third, students self-selected when to use the H&P 360 as compared with traditional note templates. Consequently, it is possible that there may have been systematic differences among patients represented in each group (eg, ability to engage, presence and number of needs), which may have biased the results. Fourth, we focused solely on initial admission H&P notes and did not include review of progress notes or discharge summaries. As a result, we may have missed instances in which psychosocial information was documented later during a patient’s hospital course. Fifth, we did not survey patients or interdisciplinary team members about their experiences with the H&P 360 and did not collect any other objective systems-level data on the impact of the H&P 360 on discharge planning or resource provision. As a result, our findings are limited by the accuracy and completeness of subI documentation. Lastly, the survey response rate was low, in part due to inclusion of students on their ICU rotation who were unlikely to utilize the H&P 360 owing to competing acute priorities. While the response rate was overall lower than ideal, the students who did complete the survey likely represented a large majority of those who utilized the EHR template.

### Conclusions

Integrating the H&P 360 framework into templated notes in the EHR is feasible, and may increase assessment of goals and perspectives for patient-engaged care and contextual factors important to prevention of rehospitalization. Uptake of the note template may be enhanced through earlier and repeated exposure, encouraging paced usage over the course of a hospitalization, and greater engagement by residents and attendings. Larger-scale implementation studies with learners and practicing clinicians, paired with robust evaluation efforts involving patients, clinicians, and interprofessional staff, are needed to better understand the complexities of implementing nonbiomedical information within EHRs and the usual flow of care.

## Appendix

Multimedia Appendix 1 Seven Domain Biopsychosocial Framework (basis for the history and physical [H&P] template).

Multimedia Appendix 2 Full history and physical (H&P) template.

Multimedia Appendix 3 Brief history and physical (H&P) template.

Multimedia Appendix 4 Student survey.

Multimedia Appendix 5 Utilization of the health and physical (H&P) 360 template by subinterns (n=13 students).

## Abbreviations

EHR: electronic health record
H&P: history and physical
ICU: intensive care unit
REDCap: Research Electronic Data Capture
subI: subintern
UC: University of Chicago

## Data Availability

Anonymized survey data are available from the corresponding author on reasonable request. The patient notes analyzed during the current study are not publicly available to protect patient anonymity.
