# Supplementary material for: Implementation of a Biopsychosocial History and Physical Exam Template in the Electronic Health Record: Mixed Methods Study
Source: JMIR Med Educ. 2023 Feb 21;9:e42364. doi: 10.2196/42364 (PMC9993233; doi:10.2196/42364)
Supplement: Multimedia Appendix 1 [file mededu_v9i1e42364_app1.doc]

**Appendix 1: Seven Domain Biopsychosocial Framework (basis for the H&P360 template)**

| **Domains** | **Relevant content areas** |
| --- | --- |
| Patient perspectives and goals | Patient understanding of health, perceived control, health goals, advance care planning |
| Mental health | Mental health conditions (depression, anxiety, etc), cognitive impairment |
| Behavioral health | Medication adherence, exercise, nutrition, substance use |
| Social support | Primary relationships, caregiver availability/capacity, domestic abuse, community relationships |
| Living environment and resources | Financial resources, insurance status, access to healthcare, housing, food security, transportation |
| Functional status | ADLs, IADLs, education, occupation, mobility devices |
| Biomedical information | Chronic and acute medical conditions |
