# Supplementary material for: Implementation of a Biopsychosocial History and Physical Exam Template in the Electronic Health Record: Mixed Methods Study
Source: JMIR Med Educ. 2023 Feb 21;9:e42364. doi: 10.2196/42364 (PMC9993233; doi:10.2196/42364)
Supplement: Multimedia Appendix 2 [file mededu_v9i1e42364_app2.doc]

**Appendix 2: Full H&P 360 template**

**MEDICAL STUDENT HISTORY AND PHYSICAL**

**@FDATE@**

**Patient:** @NAME@

**Room:** @ROOMBED@

**Medical Records Reviewed:**  {MEDICAL RECORDS REVIEWED:91000}

***SUBJECTIVE***

**CHIEF COMPLAINT/REASON FOR ADMISSION**

***

**HISTORY OF PRESENT ILLNESS**

**Biomedical problems and concerns:**

***

**Patient perception of health:**

Patient understanding of health: ***

Patient self-assessed control: ***

Patient-identified strengths: ***

Patient-identified barriers: ***

**Patient priorities and goals:**

1. ***
2. ***

**Mental health problems and concerns:**

***

**PAST MEDICAL HISTORY**

@HXPMH@

**PAST SURGICAL HISTORY**

@HXPSH@

**FAMILY HISTORY**

@FAMHX@

**SOCIAL HISTORY**

**Behavioral health** (e.g., health behaviors, medication adherence, nutrition, physical activity, substance use):

***

**Social support** (e.g., relationships, caregiver, violence)

***

**Living environment and resources** (e.g., transportation, food security, housing stability, financial resources)

***

**Function** (e.g., use of assistive device, ADL/IADLs, social and occupational functioning)

***

@SHORTSOCIALHISTORY@

**PRIOR TO ADMISSION MEDICATIONS**

@HMEDS@

**ALLERGIES AND DRUG REACTIONS**

@ALLERGY@

**REVIEW OF SYSTEMS**

**@ROSBYAGE@**

***OBJECTIVE***

**VITALS**

The patient's @VSP@

**PHYSICAL EXAMINATION**

@PHYSEXAM@

**REVIEW OF LABORATORY DATA**

**CBC:**

@LABRCNT(WBC:2,RBC:2,HGB:2,HCT:2,MCV:2,MCH:2,MCHC:2,RDW:2,PLT:2,MPV:2,PGRA:2,TDIF:2,GRANULOCYTES:2,LYMPHOCYTES:2,MONOCYTES:2,EOSINOPHILS:2,BASOPHILS:2,ABSGRANS:2,ABL:2,ABMO:2,ABE:2,ABB:2,NEUT:2,BANDS:2)@

@LABRCNT(RELY,NMET,MYEL,PROMY,BLA,ABSNEUTS,ABSBANDS,ABMM,ABMY,ABPM,ABBL)@

**BMP / Mag / Phos:**

@LABRCNT(na:2,k:2,chloride:2,co2:2,bun:2,creat:2,gluc:2,calcium:2,mag:2,phos:2,gfr:2)@

**Liver Function Tests:**

@LABRCNT(TOTPROT,ALB,BILTOTAL,BILCONJ,BILUNCONJ,AST,ALT,alkphos)@

**Coags:**

@LASTLABBRIEF(protime,ptt,inr)@

**Cardiac Biomarkers:**

@LABRCNT(cpk:*,ckmbmass:*,ckmbinterp:*,troponin:*)@

**Lipid Panel:**

@LASTLABBRIEF(CHOL,HDL,TRIG,LDLC)@

**HgbA1c:**

@LASTLABBRIEF(a1c)@

**Thyroid Function Tests:**

@LASTLABBRIEF(tsh,tt4,ft4,ft3,t3,tbg)@

**Urine Studies:**

@LASTLABBRIEF(COLOR,CLARITY,SPECGRAV,URPH,LEUKESTERASE,URNITRITE,URPROTEIN,BLOU,URGLUCOSE,RSU,URKETONES,URBILIRUBIN,UROBILINOGEN,RBCU,WBCU,HYAL,GCAST,WCAST,RCAST,ECAST,XCAST,FCAST,SQUAM,TEPI,REPI,BACTE,YEAST,UTRIC,CRYU)@

**Micro:**

@RESLTCXU@

@RESLTCXBLD@

@RESLTCXRESP@

**REVIEW OF RADIOLOGY IMAGES & OTHER DIAGNOSTIC STUDIES**

***

***ASSESSMENT & PLAN***

**@NAME@** is a **@AGE@** old **@SEX@ with *** who presents with *****

**1. Active Medical Issues**

**#***:**

**#***:**

**#***:**

**2. Chronic Medical Issues**

**#***:**

**#***:**

**#***:**

**3. Interdisciplinary Resource Needs** (E.g., self-management support, community referrals, home health, equipment needs, discharge plans)

**#***:**

**#***:**

**4. Hospitalization**

**#DVT Prophylaxis:**

Intermountain VTE Risk Stratification (Woller, et al., Am J Med. 2011:124;947-952)

Pt is at risk for DVT and should be prophylactically anticoagulated if 1 or more of the following:

- Previous VTE
- PICC
- Current Cancer
- Pt Immobile

**PPX:**{HMPPX:59}

**#Diet/Fluids:** {FEN:2207}

**#PT/OT:** {PT/OT Ordered:2208}

**#Dispo:** {Inpatient/Observation:2209}

**INTERDISCIPLINARY PLAN FOR COMMUNICATION WITH PROVIDERS**

These providers/consults services were notified of the patients admission: ***

**The attending physician will comment on the risk/complexity below.**

@ME@
