# Supplementary material for: Implementation of a Biopsychosocial History and Physical Exam Template in the Electronic Health Record: Mixed Methods Study
Source: JMIR Med Educ. 2023 Feb 21;9:e42364. doi: 10.2196/42364 (PMC9993233; doi:10.2196/42364)
Supplement: Multimedia Appendix 3 [file mededu_v9i1e42364_app3.doc]

**Appendix 3: Brief H&P 360 template**

**HPI:**

**Biomedical problems and concerns:**

***

**Patient perception of health:**

***

**Patient priorities and goals:**

***

**Mental health problems and concerns:**

***

**SOCIAL HISTORY:**

**Behavioral health** (health behaviors, medication adherence, nutrition, physical activity, substance use):

***

**Social support** (relationships, caregiver, violence)

***

**Living environment and resources** (food security, housing stability, financial resources, transportation)

***

**Function** (ADL/IADLs, social and occupational functioning, use of assistive device)

***

**ASSESSMENT AND PLAN:**

**1. Active Medical Issues**

**#***:**

**#***:**

**#***:**

**2. Chronic Medical Issues**

**#***:**

**#***:**

**#***:**

**3. Interdisciplinary Resource Needs** (E.g., self-management support, community referrals, home health, equipment needs, discharge plans)

**#***:**

**#***:**
