# Supplementary material for: Implementation of a Biopsychosocial History and Physical Exam Template in the Electronic Health Record: Mixed Methods Study
Source: JMIR Med Educ. 2023 Feb 21;9:e42364. doi: 10.2196/42364 (PMC9993233; doi:10.2196/42364)
Supplement: Multimedia Appendix 4 [file mededu_v9i1e42364_app4.doc]

**Appendix 4: Student survey**

**HP360 STUDENT SURVEY**

**AMA 4-SITE CONSORTIUM 2020 (v. 4)**

**Planned Specialty:**

**Graduation Year:** **Date:**

1. For each of the following domains of the H&P360 social history, please provide an example of a question that would be relevant to that domain:

- 1. Patient Goals:
  2. Behavioral:
  3. Social support:
  4. Living environment and resources:
  5. Functional status:

1. Please rate your level of agreement with the following statements:

| **STATEMENTS:** | **Strongly Disagree****1** | **Somewhat Disagree****2** | **Neither Agree nor Disagree****3** | **Somewhat Agree**  **4** | **Strongly Agree****5** |
| --- | --- | --- | --- | --- | --- |
| The H&P 360 changed some of the questions I ask patients during the encounter. |  |  |  |  |  |
| The H&P 360 was easy to use. |  |  |  |  |  |
| The H&P 360 took an appropriate amount of time to complete. |  |  |  |  |  |
| The H&P 360 helped me create a more comprehensive problem list |  |  |  |  |  |
| The H&P 360 added valuable information that I would not otherwise know about the patient. |  |  |  |  |  |
| The H&P 360 helped me learn to be a better clinician. |  |  |  |  |  |
| The H&P 360 helped me better understand patients’ goals |  |  |  |  |  |
| Using the H&P 360 facilitated a stronger provider-patient relationship. |  |  |  |  |  |
| 1. I was able to develop management plans that incorporated information from the H&P 360 |  |  |  |  |  |
| 1. The H&P 360 facilitated care planning that included other health professionals (e.g., social work, nursing, pharmacy, physical therapy) |  |  |  |  |  |
| 1. Elements of the H&P 360 could be incorporated into every patient interaction. |  |  |  |  |  |
| 1. Presentations using the H&P 360 were well received by my clinical team. |  |  |  |  |  |
| 1. The H&P 360 helped improve the care I provided to my patients. |  |  |  |  |  |
| 1. I plan to use the H&P 360 during other rotations. |  |  |  |  |  |

1. Name two (or more) aspects of the H&P 360 you found helpful.
2. Name two (or more) aspects of the H&P 360 you found challenging.
3. What changes would you recommend for the H&P 360?
