# Supplementary figures and images for: Implementation of a Biopsychosocial History and Physical Exam Template in the Electronic Health Record: Mixed Methods Study
Source: JMIR Med Educ. 2023 Feb 21;9:e42364. doi: 10.2196/42364 (PMC9993233; doi:10.2196/42364)

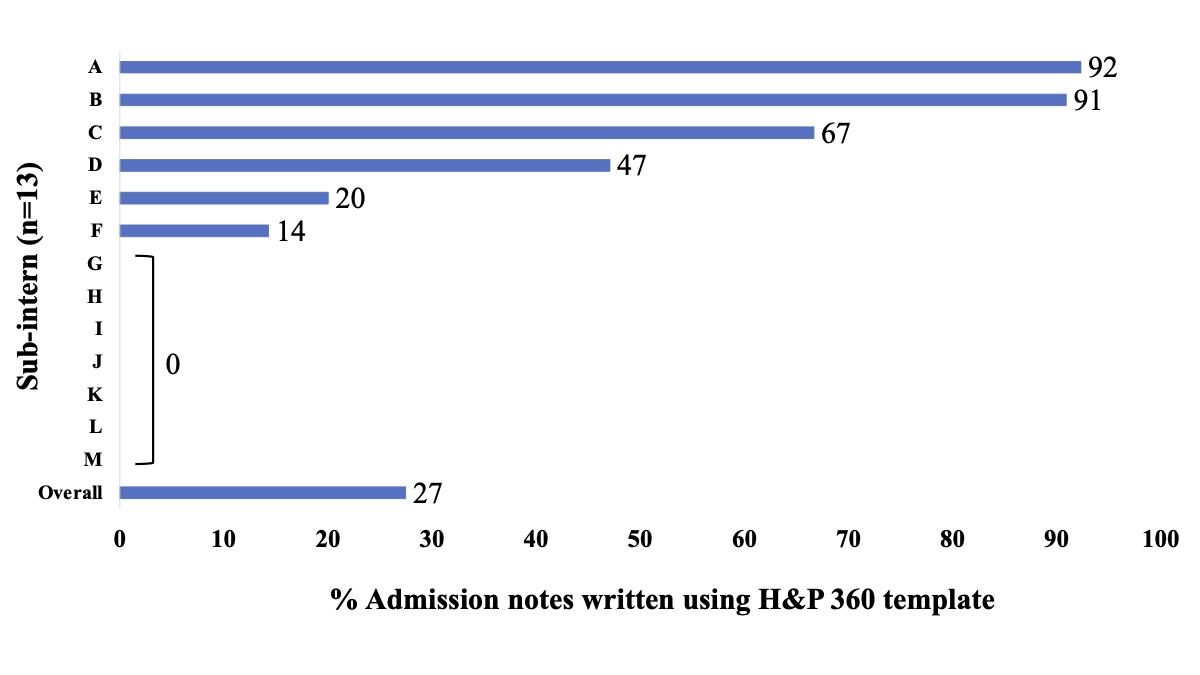

Supplement: Multimedia Appendix 5 [file mededu_v9i1e42364_app5.png]
